# CAQK, a peptide associating with extracellular matrix components targets sites of demyelinating injuries

**DOI:** 10.3389/fncel.2022.908401

**Published:** 2022-08-22

**Authors:** Charly Abi-Ghanem, Deepa Jonnalagadda, Jerold Chun, Yasuyuki Kihara, Barbara Ranscht

**Affiliations:** Center for Genetic Disorders and Aging, Sanford Burnham Prebys Medical Discovery Institute, La Jolla, CA, United States

**Keywords:** peptide targeting, extracellular matrix, mouse demyelination models, tenascins, myelin repair

## Abstract

The destruction of the myelin sheath that encircles axons leads to impairments of nerve conduction and neuronal dysfunctions. A major demyelinating disorder is multiple sclerosis (MS), a progressively disabling disease in which immune cells attack the myelin. To date, there are no therapies to target selectively myelin lesions, repair the myelin or stop MS progression. Small peptides recognizing epitopes selectively exposed at sites of injury show promise for targeting therapeutics in various pathologies. Here we show the selective homing of the four amino acid peptide, cysteine-alanine-lysine glutamine (CAQK), to sites of demyelinating injuries in three different mouse models. Homing was assessed by administering fluorescein amine (FAM)-labeled peptides into the bloodstream of mice and analyzing sites of demyelination in comparison with healthy brain or spinal cord tissue. FAM-CAQK selectively targeted demyelinating areas in all three models and was absent from healthy tissue. At lesion sites, the peptide was primarily associated with the fibrous extracellular matrix (ECM) deposited in interstitial spaces proximal to reactive astrocytes. Association of FAM-CAQK was detected with tenascin-C although tenascin depositions made up only a minor portion of the examined lesion sites. In mice on a 6-week cuprizone diet, FAM-CAQK peptide crossed the nearly intact blood-brain barrier and homed to demyelinating fiber tracts. These results demonstrate the selective targeting of CAQK to demyelinating injuries under multiple conditions and confirm the previously reported association with the ECM. This work sets the stage for further developing CAQK peptide targeting for diagnostic and therapeutic applications aimed at localized myelin repair.

## Introduction

Myelin, a multilayered, lipid-rich membrane sheath formed by oligodendrocytes in the central nervous system (CNS), enables rapid nerve impulse conduction and sustains the functional integrity of neurons and their encircled axons. In multiple neurodegenerative diseases, compromised myelin and impaired nerve functions are closely interlinked. In multiple sclerosis (MS), peripherally activated T-cells and macrophages break through the blood-brain barrier and infiltrate the brain parenchyma causing local inflammation (Mapunda et al., 2022), thus activating a host of tissue reactions that proceed to destroying the myelin. Myelin injury and death of oligodendrocytes impair nerve functions and lead to neurodegeneration with progressive patient disability. Although current MS therapies are efficient in altering or suppressing the immune response, there are no treatments to repair damaged myelin and thus protect nerve functions. Oligodendrocyte progenitor cells (OPC) residing near MS lesion sites retain a limited myelin repair capacity during the early stages of the disease, however, they lose this vital ability with disease progression (Franklin and Goldman, 2015). The cause for remyelination failure is multi-factorial and includes lesion-associated factors that impinge on OPC proliferation, inhibit progenitor migration into lesion sites, or halt OPC maturation, and myelin formation (Galloway et al., 2020). Innovative approaches are needed to identify novel therapies that enable OPC-mediated regeneration of myelin during disease advancement or increase the resilience of myelin to damage.

Significant progress has been made *in vitro* and in animal models in identifying molecules and mechanisms that promote or inhibit remyelination (Franklin, 2002; Franklin and Ffrench-Constant, 2017). Amongst the latter, extracellular matrix (ECM) components are dynamically remodeled during injury and their interactions with surrounding cells exposing new epitopes or receptors induce injury-related pathological pathways (Zimmermann and Dours-Zimmermann, 2008; de Jong et al., 2020; Srivastava et al., 2020; Su et al., 2021). Upregulation of ECM components, including chondroitin sulfate proteoglycans (CSPGs), tenascins, and fragmented hyaluronan, block steps in OPC differentiation and myelination and are implicated in contributing to remyelination failure (Czopka et al., 2010; Karus et al., 2016; Srivastava et al., 2020; Su et al., 2021). Accordingly, studies in mouse models show that CSPG synthesis block or pharmacologically induced clearance promotes remyelination (Lau et al., 2012; Karus et al., 2016; Keough et al., 2016). Interventions with inhibitory tenascin-C deposition reduce susceptibility to inflammatory demyelinating experimental autoimmune encephalomyelitis (EAE) and improve remyelination in mice (Momcilovic et al., 2017; Bauch and Faissner, 2022). Thus, approaches to hinder the deposition of pathological ECM components or to neutralize its adverse effects have gained interest in approaching myelin repair.

With better molecular understanding of myelin destruction and repair processes, the next critical step is translating these findings into remyelination therapies. This entails the development of therapeutics effective in stimulating the remyelination processes and concentrating them at effective doses at sites of myelin damage. Systemic administration often requires high therapeutic concentrations that can lead to undesired side effects in patients. A further challenge is drug delivery across the blood-brain barrier (BBB) that limits access to the CNS (Daneman and Prat, 2015). A promising approach to overcome these limitations is the use of small peptides that target epitopes selectively exposed at myelin injuries. Differentially expressed molecules at lesion sites can serve as docking sites for concentrating drug conjugates or cargo-loaded nanoparticles, and thus increase the efficacy of targeted therapeutics while limiting side effects (Ruoslahti et al., 2010). Peptide targeting is emerging as a promising therapy for a variety of diseases (reviewed in Wang et al., 2022). In cancer, peptides deliver agents to unfold local actions that change signaling pathways or serve as agonists or antagonists for cancer-related receptors (Olson et al., 2009; Thundimadathil, 2012; Marqus et al., 2017; Ruoslahti, 2017; Scodeller and Asciutto, 2020). Small peptides are often able to cross the blood-brain barrier raising a potential additional advantage for CNS targeting (Islam et al., 2021).

Aiming to identify peptides for targeting traumatic brain injuries (TBI), Rouslahti and colleagues conducted a systemic *in vivo* peptide phage display library screen that identified a novel four amino acid peptide, cysteine-alanine-glutamine-lysine, referred to as CAQK (Mann et al., 2016). This peptide selectively associates with focal and impact-induced brain lesions while not interacting with healthy brain and other tissues (Mann et al., 2016). Traumatic brain injury (TBI) lesions feature a myriad of cellular alterations, including the breakdown of the BBB, immune cell infiltration, activation of astrocytes and microglia, axon- and neuron degeneration, extracellular matrix remodeling, demyelination and oligodendrocyte death (George and Geller, 2018; Qin et al., 2021). Many of these key cellular pathologies in TBI are manifest in MS and other diseases associated with myelin destruction (Su et al., 2021). We thus speculated that CAQK peptide may also target sites of demyelination and potentially prove useful in advancing therapies aimed at achieving myelin repair. We here examined the homing of CAQK tetrapeptide to sites of myelin damage in mouse models of acute, immune-mediated, and toxic demyelination. We report selective targeting of CAQK to injured but not intact myelin in the three demyelination models and thus open prospects for exploring the potential utility of CAQK towards myelin repair.

## Materials and Methods

### Materials

Peptides were synthesized with an N-terminal fluorescein amine (FAM) tag and provided at >90% purity by (Genscript). Lyophilized aliquots of ~3–4 mg were stored at −80°C and reconstituted with sterile phosphate-buffered saline (PBS) for use in mice. Lysolecithin was from Sigma (Cat# L4129) and cuprizone from Thermo Fisher Scientific (Cat# 370-81-0).

### Mice and mouse models of demyelination

All animal procedures were conducted in accordance with Institute Animal Care and Use Committee guidelines at Sanford Burnham Prebys Medical Discovery Institute (La Jolla, CA). The C57BL6/J mouse strain was used in all experiments. Mice constitutively expressing the red fluorescent tdTomato reporter in oligodendrocytes (in text CNP1-TdTom mice) were generated by crossing CNP1-Cre transgenic mice (Lappe-Siefke et al., 2003) with the Rosa26-tdTomato reporter (Jax # 007909) and used in some experiments.

### Lysolecithin-induced focal demyelination

Focal demyelination was induced by stereotaxic injection of 1 μl of 1% lysolecithin solution into the right ventral white matter of 8–10-week-old male mouse spinal cords under ketamine/xylazine anesthesia (Arnett et al., 2004; Bielecki et al., 2016). Injections were made after piercing the dura mater with an 18G needle into the space between T12 and T13 and inserting a fine-tipped glass capillary connected to a Hamilton syringe mounted on an infusion pump run at a rate of 0.2 μl per minute over 5 min. The capillary was kept in place for at least 2 min to allow the solution to diffuse before retraction of the capillary and incision closure. Sham-operated mice were injected with vehicle (PBS). Animals were allowed to recover from surgery and were used for experimentation 24 h and 5 days after LPC injection.

### Experimental autoimmune encephalomyelitis (EAE)

Robust EAE was reproducibly established in 8–12-week old female mice by immunizing groups of 10 or 20 animals with 200 μg MOG_35–55_ peptide in combination with 200 mg *Mycobacterium tuberculosis* emulsified in complete Freund’s adjuvant (CFA; EAE kit 2110; Hooke Laboratories, Lawrence, MA, United States) according to the manufacturer’s protocol. Pertussis toxin (PTX; 80 ng in PBS) was administered intraperitoneally (i.p.) at the time of induction 0 and 24-h later. Mice in the control group were injected with PTX only. Females from the control and the experimental group were housed together in a cage. Starting at post-induction day 5 (PID5) and ending on PID 28, mice were scored daily for clinical symptoms on the established rating scale (Kihara et al., 2005; Jonnalagadda et al., 2021). Mice were weighed and scored daily for signs of progressive paralysis: 0—no abnormalities; 0.5—mild loss of tail tone; 1.0—complete loss of tail tone; 1.5—mildly abnormal gait and difficulty in righting; 2.0—abnormal gait and hindlimb weakness; 2.5—beginning hindlimb paralysis; 3.0—complete or almost complete hindlimb paralysis; 3.5—paralysis and inability to upright body; 4.0—hindlimb paralysis and forelimb weakness or paralysis; 4.5—paralysis without an attempt to move around the cage; 5.0—moribund or dead. The scoring was performed blinded to the experimenter.

### Demyelination with cuprizone

C57BL/6J 8-week-old male mice were maintained for 6 weeks on a ground chow without or supplemented with 0.2% cuprizone (Bis (cyclohexanone) oxaldihydrazone 98%; Thermo Fisher Scientific, Cat# 370-81-0). Mice were weighed every other day and the cuprizone-fed group showed lower body weights compared to control mice on a normal chow consistent with the effectiveness of the supplement (Tagge et al., 2016).

### Peptides

In all experiments, FAM-CAQK or FAM-control scrambled (FAM-ACKQ) peptides were administered at 100 nmoles/100 μl in PBS into the tail vein or the plexus retroborbitalis. After 60 min in the circulation, animals were anesthetized by inhalation of isofluorane (VetOne, Cat#502017). Circulating FAM-peptides were removed from the bloodstream by perfusion with PBS. Spinal cords were isolated and drop fixed in 4% paraformaldehyde (PFA; Sigma Aldrich, Cat#P6148) in PBS overnight. For harvesting brain cortices, animals were perfused with 4% PFA in PBS. Tissue was postfixed overnight at 4°C in 4% PFA. All tissues were cryoprotected by immersion into 30% sucrose solution 48 h before freezing and then embedded in optimal cutting temperature (OCT) compound (Scigen Cat# 4586) on dry ice/isopentane/methyl pentane at −78°C. Tissues were kept frozen at −20°C until use.

### Processing and immunohistochemistry of spinal cord tissue

Spinal cords from LPC injured or sham-operated mice were cut in cross-section at the level of the injury site. Spinal tissue from mice with EAE or controls was partitioned into four pieces and embedded en block for simultaneous cutting at different levels. Crosssections were cut on a cryostat at 12 μm and collected on Fisherbrand^TM^ Superfrost^TM^ Plus microscope slides (Fisher Scientific Cat#12-550-15). Staining was performed essentially as previously described (Colakoglu et al., 2014). Unspecific binding sites were blocked by sequential incubation with 1% glycine and blocking solution (5% BSA, 2% normal donkey serum, 0.02% Triton X100) for 15 min and 1 h, respectively. Primary antibodies diluted in blocking solution were applied overnight at 4°C in a humid chamber. Slides were washed three times with PBS before being incubated with species-specific secondary antibodies coupled to either Alexa-488, Alexa-594, or Alexa-647 in Tris-buffered saline with 0.02% Triton X100 (TBST) for 1 h at room temperature. After three washes in PBS, slides were counterstained with blue-fluorescent nuclear DNA stain (DAPI) and mounted using Fluoromount G. Staining was examined on a Zeiss LSM 710 confocal microscope. The following primary antibody reagents were used: Rabbit anti-fluorescein/Oregon Green (Thermo Fisher Scientific, Cat#A-889, RRID:AB_221561), goat anti-FITC (Thermo Fisher Scientific, Cat# PA1-26793, RRID:AB_794297), rat anti-MBP aa 82-87 (Millipore, Cat# MAB386, RRID:AB_94975), rabbit anti-Olig2 (Millipore, Cat# AB9610, RRID:AB_570666), mouse mAB anti-CC1 (Millipore Cat# OP80, RRID:AB_2057371), rat anti-GFAP monoclonal antibody 2.2B10 (Thermo Fisher Scientific Cat# 13-0300, RRID:AB_2532994), rabbit anti-Iba1 (Wako Cat#019-19741, RRID:AB_839504), rat anti-tenascin-C (R&D Systems, Cat# MAB2138, RRID:AB_2203818), and goat anti-tenascin-R (R&D Systems, Cat# AF3865, RRID:AB_2207009). Cross absorbed secondary antibodies were from Thermo Fisher Scientific: donkey anti-rabbit Alexa 488 (Cat# A21206, RRID:AB_2535792); donkey anti-goat Alexa 488 (Cat# A32814TR; RRID: AB_2866497); donkey anti-rat Alexa 594 (Cat# A-21209; RRID:AB_2535795); goat anti-rabbit Alexa 594 (Cat# A11037; RRID:AB_2534095); goat anti-mouse Alexa 594 (Cat# A11005; RRID:AB_2534073); goat anti-rat Alexa 647 (Cat# A-21247, RRID:AB_141778); donkey anti-rabbit Alexa 647 (Cat# A-31573, RRID:AB_2536183); goat anti-mouse Alexa 647 (Cat# A-21235, RRID:AB_2535804). Negative signals in controls without primary antibodies and the use of primary antibodies of inappropriate species established specificity of the secondary antibodies.

### Staining of brain tissue

Coronal sections (30 μm; Leica cryostat CM3050S) were cut in anterior to posterior direction starting at the level of bregma -1.58 mm. To estimate the status of myelination, brain sections were mounted onto gelatin-coated slides for staining with Black Gold II (Biosensis, Thebarton, SA, Australia; Cat #TR-100-BG). Free floating immunofluorescence staining was performed to detect different neural cell types and extracellular matrix proteins. Briefly, after washing the brain sections in PBS, the FAM signal was amplified with an anti–FITC polyclonal antibody (Thermo Fisher Scientific, Cat# PA1-26793, RRID:AB_794297) or fluorescein/Oregon green polyclonal antibody (Thermo Fisher Scientific, Cat# A-889; RRID:AB_221561). Primary antibodies used were chicken anti-GFAP (Neuromics, Cat#CH22102, RRID:AB_10014322), rabbit anti-MBP (Abcam, Cat# ab-40390, RRID:AB_1141521), rabbit anti-Iba1 (Wako, Cat# 019–19741, RRID:AB_839504), goat anti-tenascin R (R&D Systems, Cat# AF3865, RRID:AB_2207009), and rat anti-tenascin C (R&D systems, Cat# MAB2138, RRID:AB_2203818) in 5% normal donkey serum (Jackson Immuno Research Cat# 017-000-121) at room temperature overnight. The tissue was washed thrice in PBS the next day for 5 min each and incubated with the corresponding secondary antibody (goat anti-chicken Alexa Fluor 568, Thermo Fisher Scientific, Cat#A-11041, RRID:AB_2534098; donkey anti–rabbit Alexa Fluor 568 Invitrogen, Cat#A-10042, RRID:AB_2534017; goat anti-rat IgM Alexa Fluor 647, Thermo Fisher Scientific, Cat# A21248, RRID:AB_2535816; donkey anti-goat Alexa Fluor 647, Thermo Fisher Scientific, Cat# A21447, RRID:AB_2535864) in 0.3% TritonX-100/PBS for 1.5 h at room temperature in the dark. Care was taken to minimize exposure to light from this step onward. After washing thrice in PBS, the sections were mounted onto gelatin-coated slides and coverslipped with a mounting medium containing DAPI (Vectashield, Cat#H-1500).

### Imaging

Sections with fluorescent labels were imaged on the Zeiss LSM 710 NLO Multiphoton microscope. Histological brain sections were analyzed using a Keyence microscope (BZ-X-800 series). ImageJ version 1.52q^1^ was used for image processing.

### Data reproducibility

For each time point and condition, 3–5 animals were used, and from each animal 2–4 sections were examined to obtain results.

## Results

### Mouse models of demyelination

To probe the ability of CAQK peptide to associate with sites of demyelination injury, we employed multiple mouse models that reflect different aspects of acute and chronic demyelination (Denic et al., 2011; Ransohoff, 2012). Rapid focal demyelination with immune cell infiltration and a compromised blood-brain barrier was induced by a local lysophosphatidylcholine (LPC; lysolecithin) injection into the spinal cord. In this model, acute spatiotemporally precise damages to the lipid-rich myelin membrane sheaths are observed at the injection site as early as 24 h with continuing demyelination for up to 7 days after injury (Hall, 1972; El Waly et al., 2014). In the toxin-induced cuprizone model, demyelination occurs in mice fed the cuprizone diet with a highly reproducible time course and well-defined mechanisms in the corpus callosum and more variable results in other fiber tracts (Skripuletz et al., 2011; Praet et al., 2014). Cuprizone is a copper chelator that induces mitochondrial toxicity and death of oligodendrocytes. After 6-weeks on the cuprizone diet, mice show strain-reproducible demyelination in the brain with well-characterized spatiotemporal cellular changes in the corpus callosum in the presence of a nearly closed blood-brain barrier (Praet et al., 2014). Finally, chronic inflammatory demyelination that most closely mirrors features of human MS occurs in the EAE model (Constantinescu et al., 2011; Hasselmann et al., 2017). Here, peripheral administration of myelin protein peptides results in the activation of peripheral T-cells that transgress the blood-brain barrier where they induce inflammatory myelin damage accompanied by neurodegeneration and gliosis with simultaneous counteracting anti–inflammatory processes and remyelination. EAE mice develop ascending paralysis after EAE induction with the most severe clinical manifestations in the spinal cord permitting the monitoring of disease severity. Utilizing these models allowed us to assess CAQK peptide association in the demyelinated brain and spinal cord under different pathophysiological conditions.

### CAQK peptide homes to sites of focal demyelination inflicted by acute lysolecithin injection

**Figure 1:**
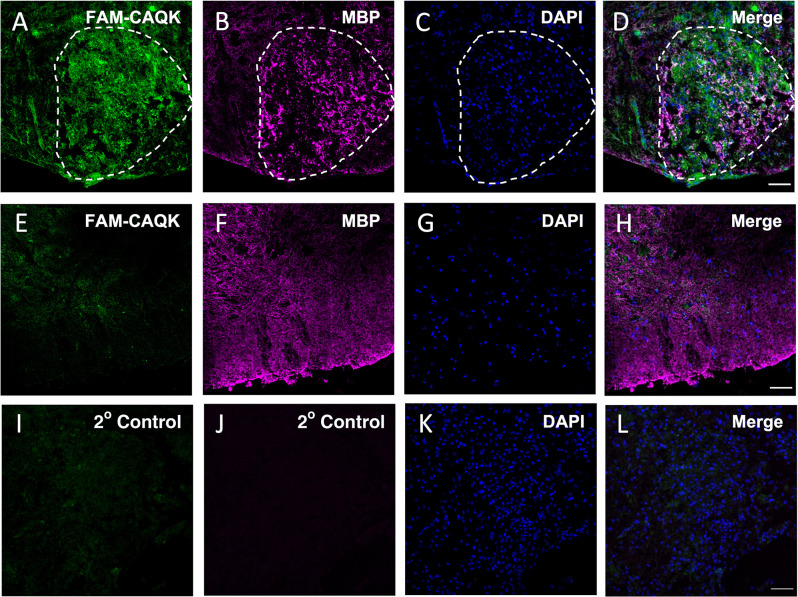
CAQK homes to lysolecithin-induced focal lesions in the spinal cord. FAM-CAQK (green) accumulates at lesion sites 5 days after LPC injection (**A–D**, LPC). The lesion area outlined by white dashes was identified by disorganized MBP staining (**B**, magenta) and the increased density of mononucleated DAPI^+^ cells (blue in **C,K**) compared to healthy tissue (blue in **G**). FAM-CAQK peptide **(E)** does not accumulate in white matter (**F**, MBP, magenta) in sham-operated, PBS-injected spinal cord tissue (**E–H**, sham). Panels **(I–L)** show the corresponding secondary antibody controls around a lesion site in LPC-injected tissue. *N* = 3–4 mice per group. Scale bar 50 μm.

Acute focal demyelination induced by stereotactic injection of lysolecithin results in a defined injury site with an open blood-brain barrier like in the TBI model plus has high selective toxicity to oligodendrocytes and myelin while leaving neurons and axons largely intact (Blakemore and Franklin, 2008). Demyelination is detectable within 24 h and reaches a maximum by 7 days post LPC injection after which remyelination occurs (El Waly et al., 2014). We employed this rapid model to test the hypothesis that CAQK peptide homes to injured myelin. A unilateral injection of 1% lysolecithin was made into the ventral funiculus of the mouse spinal cord to induce the lesion. FAM-CAQK was administered intravenously at a concentration of 100 nmoles/100 μl at 24 h or 5 days post-injury and allowed to circulate for 1 h before perfusion and tissue harvesting. Sites of demyelinating injury correlate with the accumulation of DAPI^+^ stained mononuclear cells (Luo et al., 2014; Bielecki et al., 2016; Lozinski et al., 2021). We utilized this characteristic to localize the lesion area and detect the FAM-CAQK label in the ventral spinal cord white matter near the injection site. As illustrated for the 5-day post lesion time point, we observed the accumulation of FAM-CAQK peptide at the injury site identified by an increased density of mononucleated DAPI^+^ cells and fragmented myelin stained for myelin basic protein (MBP; Figures 1A–D). The peptide was not detected in the spinal cords of control sham-operated (PBS injected) mice showing regular densities of DAPI^+^ cells (Figures 1E–H), and secondary antibody controls at a DAPI-dense lesion site showed no signals (Figures 1I–L). Similar results were obtained for the 24 h post lesion time point (not shown). These observations supported the ability of CAQK to home to lesion sites in an established mouse demyelination model and encouraged further studies in long-term mouse models of demyelination.

### CAQK homes to the demyelinated corpus callosum in the cuprizone model

To attain further evidence for the targeting of CAQK peptides to sites of demyelinating injury, we employed additional mouse models of established demyelination. First, since acute LPC-induced demyelination is induced by inflicting an open wound that disrupts the blood-brain barrier and leads to immune cell accumulation, we sought to investigate CAQK targeting in a model of demyelination that is independent of peripheral immune cell invasion. Feeding mice a diet containing the neurotoxin cuprizone leads to myelin loss in central white matter tracks (Praet et al., 2014). Demyelination extends to multiple brain areas, including the cortex and hippocampus, and is well-defined and highly reproducible in the corpus callosum in the C57Bl6 mouse strain (Skripuletz et al., 2008; Schmidt et al., 2013). Although early alterations of the BBB have been noted (Skripuletz et al., 2011; Berghoff et al., 2017), the blood-brain barrier is intact in C57BL/6 mice on the cuprizone diet for 6 weeks allowing studies of demyelination in the absence of a peripheral immune response. Thus, to characterize CAQK peptide targeting under non-inflammatory conditions and an intact (or minimally altered) BBB, we fed 8-week-old mice a 0.2% cuprizone diet for 6 weeks (Praet et al., 2014), and then assessed homing of FAM-CAQK peptide to demyelinating fiber tracts of the corpus callosum.

**Figure 2:**
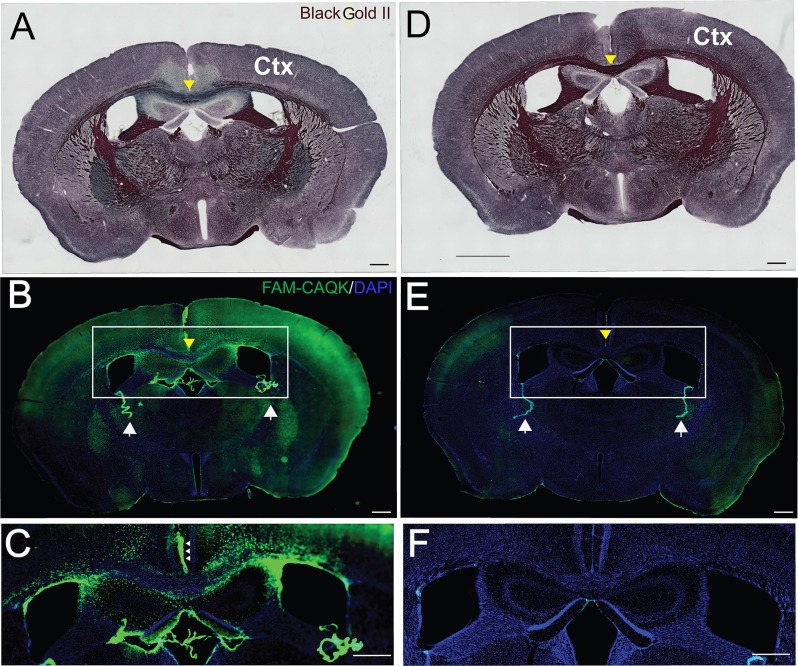
CAQK peptide targets areas of demyelination in the cuprizone model. Histological Black Gold II staining detects myelin in coronal brain sections of **(A)** FAM-CAQK peptide-treated cuprizone treated mice and **(D)** naïve mice. Demyelination is indicated by the reduction of Black Gold II labeled myelin in multiple brain areas **(A)** in mice on the cuprizone diet compared to **(D)** control mice on a normal diet. Note in particular the reduced black myelin signal in the corpus callosum in **(A)** (yellow arrowhead) compared to **(D)**; images at 10× magnification. **(B)** FAM-CAQK peptide (green) targets the demyelinated areas including the cortex (Ctx) in cuprizone-treated mice, and in **(E)** is absent from myelin in the naïve brain; images at 20× magnification with identical exposure time. In both conditions, circulating peptide associates with the choroid plexus (white arrows in **B,E**) that separates the blood from cerebral spinal fluid. The framed regions with the centered corpus callosum (yellow arrow) are shown enlarged in **(C,F)**, correspondingly. Ependymal cells facing the cerebral spinal fluid (small arrows in **C**) harbor the peptide. DAPI staining (blue) demarcates cellular nuclei in **(B,C,E,F)**. Scale Bar = 500 μm.

The cuprizone diet produced pronounced demyelination, most evident by the reduction in Black Gold II staining in the corpus callosum (Figure 2A). For comparison, the full extent of myelination is shown in naïve mice fed a normal diet (Figure 2D). Consistent with the reported loss of myelin from several brain areas in mice on the 6-week cuprizone diet, FAM-CAQK peptide was detected in cortical and hippocampal areas and the corpus callosum (Figures 2B,C). In contrast, the peptide did not associate with normal healthy myelin in the brains of naïve mice (Figures 2E,F).

**Figure 3:**
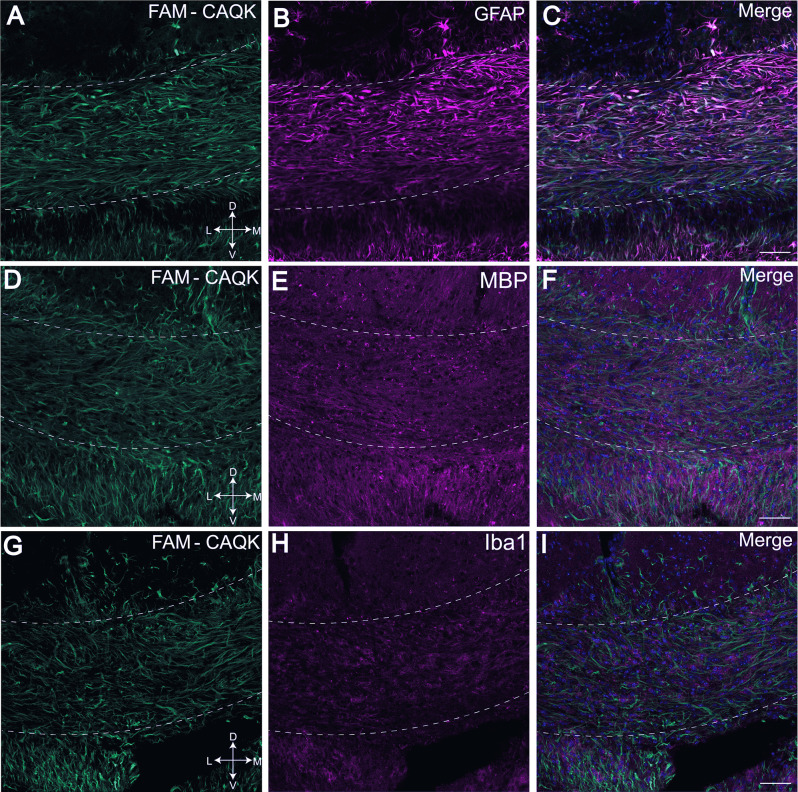
FAM-CAQK peptide associates with reactive astrocytes in corpus callosum during cuprizone-mediated demyelination. FAM-CAQK peptide is targeted to the demyelinating crossing fiber tracts in the corpus callosum (hatched lines) in mice after 6 weeks on the cuprizone diet (**A,D,G**, green). Peptide association (in **C**) is prominent with GFAP+ reactive astrocytes (**B**, magenta). Staining for MBP+ myelin (**E**, magenta) and microglial Iba1 (**H**, magenta) is marginal at the 6-week feeding point and no association of FAM-CAQK is evident; merged images **(C,F,I)** respectively. D, dorsal; V, ventral; L, lateral; M, medial. Scale bar = 50 μm.

To determine if CAQK associates with specific structures within the demyelinated corpus callosum, brain sections from peptide-treated mice fed the cuprizone diet were stained for cell type and myelin specific markers, including glial fibrillary protein (GFAP) for astrocytes (Figure 3B), MBP for myelin (Figure 3E) and ionized calcium binding adaptor molecule 1 (Iba1) for resident microglia (Figure 3H). Images taken from the medial corpus callosum show FAM-CAQK peptide staining (Figures 3A,D,G) on fibrous extracellular material associated with GFAP+ reactive astrocytes that are highly upregulated in this area (Figures 3B,C). FAM-CAQK signal also extended beyond this region to include cells with intermediate to low GFAP expression around fibers of the fornix and the dorsal hippocampal commissure (Figure 3C). Consistent with published work analyzing myelin after 6 weeks on the cuprizone diet, myelin was largely lost as indicated by a modest staining signal for MBP (Figures 3E,I; see Praet et al., 2014). Little if any of the FAM-CAQK signal overlapped with the MBP+ staining. Iba1+ microglia (Figure 3H) that are sparse at the 6-week cuprizone feeding timepoint (Praet et al., 2014) also did not show FAM-CAQK peptide association. For controls, mice fed a normal diet did not accumulate FAM-CAQK peptide in the normal myelinated corpus callosum (Supplementary Figure 1).

**Figure 4:**
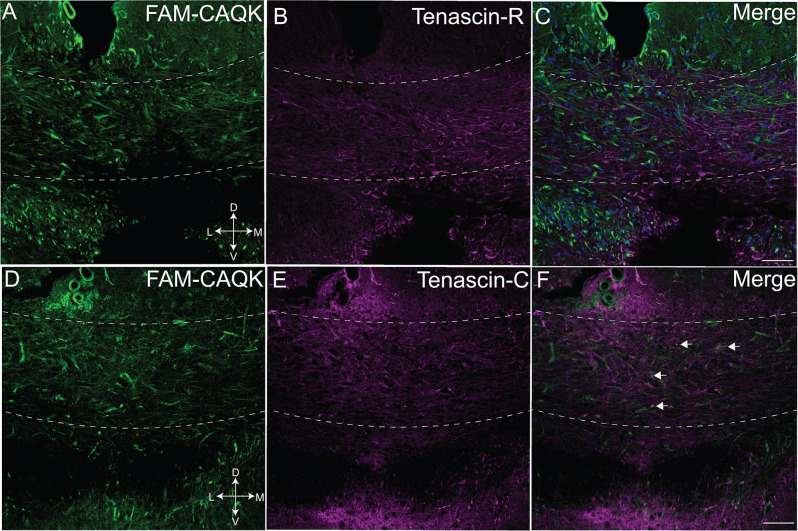
Marginal CAQK peptide association with tenascins in the cuprizone model. Confocal images from the demyelinating corpus callosum of mice on a 6-week cuprizone diet show FAM-CAQK peptide (**A,C**, green) in complementary distribution with tenascin-R (**B**, magenta); merged images in **(C)**. FAM-CAQK peptide **(D)** shows marginal association (white puncta pointed out by arrows in **F**) with tenascin-C (**E** magenta). DAPI-staining (blue) indicates cell nuclei in **(C,F)**. D, dorsal; V, ventral; L, lateral; M, medial. Scale bar = 50 μm.

Finally, as FAM-CAQK was reported to associate with extracellular matrix components in a mouse model of traumatic brain injury (Mann et al., 2016), we tested its association with extracellular matrix proteins in the cuprizone model. Tenascin-C and tenascin-R, components of the ECM produced at CNS injury sites mainly by reactive astrocytes are dynamically remodeled during demyelinating injury (Zhao et al., 2009) and were chosen for analysis. Examination of FAM-CAQK association with tenascin-R in the corpus callosum revealed minimal, if any overlap (Figures 4A–C). The images indicate that peptide association within the demyelinated corpus callosum was strongest in areas of low tenascin-R deposition (Figure 4C). Association of FAM-CAQK (Figure 4D) with tenascin-C (Figure 4E) that was weakly expressed in the demyelinated corpus callosum at the examined timepoint was restricted to only a few puncta (Figures 4D–F). These results illustrate the ability of FAM-CAQK peptide to home from the circulation through a largely intact BBB to areas of demyelination where it strongly associates with reactive astrocytes surrounded by fibrous extracellular material. The timepoint of our study thus did not yield convincing evidence for the association of the peptide with ECMs of the tenascin family and suggested targeting epitopes on yet unidentified molecules.

### CAQK targets demyelinating regions in the spinal cord in the chronic EAE model

**Figure 5:**
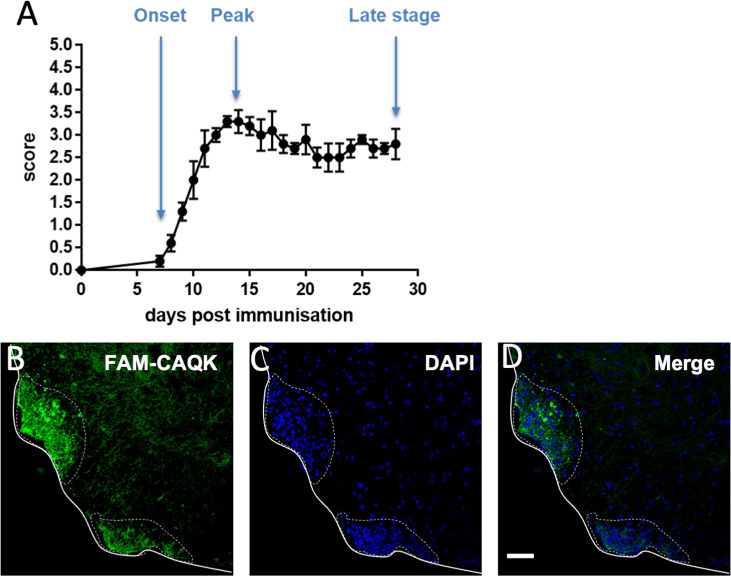
CAQK homes to demyelinating lesions in the mouse model of experimental autoimmune encephalomyelitis (EAE). **(A)** Disease scores over a 28-day period show EAE progression with an average 7-day onset and a 14-day peak. Control mice show no disease. **(B)** FAM-CAQK (green) accumulates at lesion sites in the lumbar spinal cords of mice with EAE. Lesions are indicated by clusters of DAPI-stained nuclei (blue) in **(C)** representing invasions of peripheral inflammatory cells, prominently T-cells and macrophages, into the spinal cord parenchyme. Merged images are shown in **(D)**. Continuous line delimitates the spinal cord tissue. The dotted line indicates area of active EAE lesion. Scale bar: 50 μm.

The homing of CAQK peptide in toxin-induced demyelination models prompted further investigations of CAQK targeting under pathophysiological conditions that are closely related to human MS. The chronic mouse EAE model and human MS share several critical features: In both conditions, peripherally activated immune cells infiltrate the brain parenchyma where they cause an amplified immune response that results in tissue damage, demyelination, gliosis and ultimately axon loss. In the widely used EAE model, tissue deficits in the spinal cord correlate with the formation of mononucleated cell clusters and progressive paralysis in experimental animals (Voskuhl et al., 2009; Levy-Barazany and Frenkel, 2012; Hasselmann et al., 2017). We reproducibly induced EAE with an average onset of 7–8 days and peak at 14–15 days by injection of MOG_35-55_ peptides in groups of 10 or 20 mice (Figure 5A). Focal perivascular lesions in the spinal cords of experimental mice were indicated by clusters of infiltrating DAPI-stained mononucleated cells (Figures 5B–D). CAQK homing was assessed by intravenous injection of FAM-CAQK peptide at disease onset (score 1), peak (score 3–3.5), and at late stages (28 days). FAM-CAQK showed robust targeting to lesion clusters of DAPI^+^ cells indicating immune cell invasion into the outer rim of the spinal cord parenchyma. In Figure 5, taken from a mouse at peak score, FAM-CAQK peptide targeting is shown in two lesions identified by accumulation of DAPI-stained nuclei in ventral white matter (Figures 5B–D). No peptide is seen in surrounding healthy spinal cord tissue. Similar results were obtained at EAE onset (score 1) and at late stages (28 days post-immunization, data not shown). Control mice injected with PTX only did not develop EAE and did not indicate targeting of FAM-CAQK peptide into the CNS (not shown).

### CAQK associates in proximity of cells located at lesion sites in the EAE model

**Figure 6:**
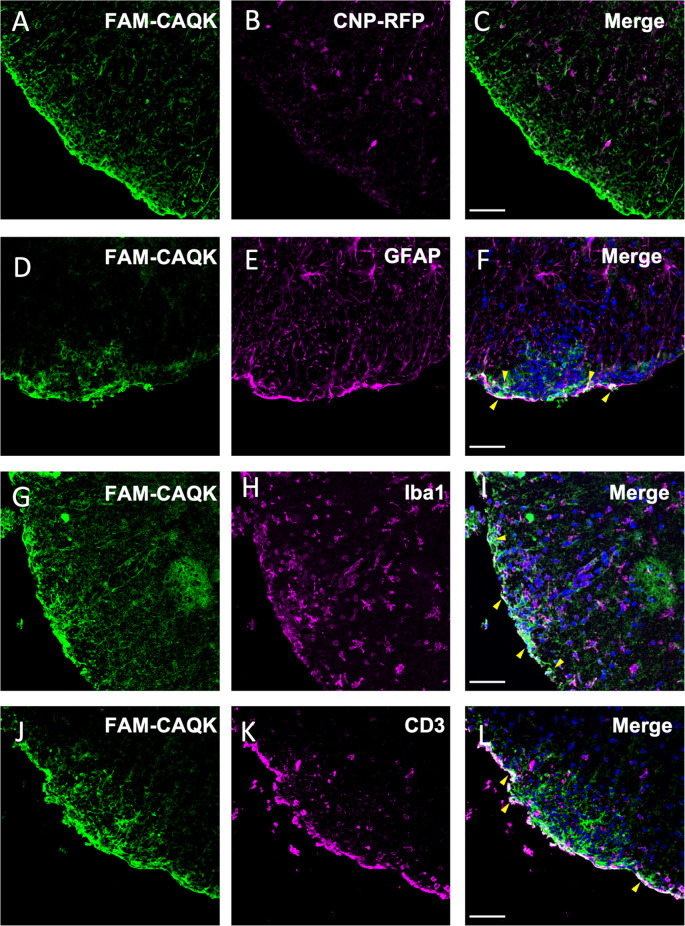
Association of CAQK with invading immune cells and reactive astrocytes at lesions in the EAE model. Fluorescent FAM-CAQK (green in **A,D,G,J**) homes to lesion sites identified by DAPI^+^ cell clusters (blue in **C,F,I,L**) after intravenous application at disease peak. The peptide does not associate with oligodendrocytes (**B**, magenta) genetically labeled in CNP1-Cre;Rosa26-tdTomato transgenic mice. Merged images in **(C)**. At the gross level, CAQK peptide associates with cells characterizing lesion sites, including reactive astrocytes (GFAP, **E**, magenta), microglia (Iba1, **H**, magenta) and infiltrating immune cells (CD3, **K**, magenta). Merged images are shown in **(C,F,I,L)**, and overlaps indicated in white color are pointed out with yellow arrowheads in **(F,I,L)**. Scale bars: 75 μm.

As CAQK is associated with demyelinating lesions in an amorphous pattern, we next queried by double labeling whether the peptide targets specific cell types. Spinal cord sections from EAE mice treated at disease onset (score 1) with FAM-CAQK peptide were used for analyses. Peptide association with oligodendrocytes was assessed in mice expressing the fluorescent tdTomato reporter in transgenic CNP1-Cre mice (Zhao et al., 2009). In contrast to observations in the TBI model (Mann et al., 2016), oligodendrocytes showed no or only a rare association with CAQK peptide (Figures 6A–C). However, we detected CAQK in association with reactive astrocytes and cells that make up the immune response. Examination of spinal cord sections revealed a gross overlap of FAM-CAQK with GPAP+ reactive astrocytes at lesions sites at the tissue rim (Figures 6D–F). Staining for microglial cells labeled with anti-Iba1 antibody (Figures 6G–I) or for CD3+ immune cells (Figures 6J–L) similarly showed partial overlap with the FAM-CAQK peptide signal. These results suggest that in the inflammatory demyelination EAE model, FAM-CAQK peptide targets lesions and localizes in proximity to infiltrating immune cells, microglia, and reactive astrocytes.

### CAQK associations with tenascins in EAE demyelinating lesions

**Figure 7:**
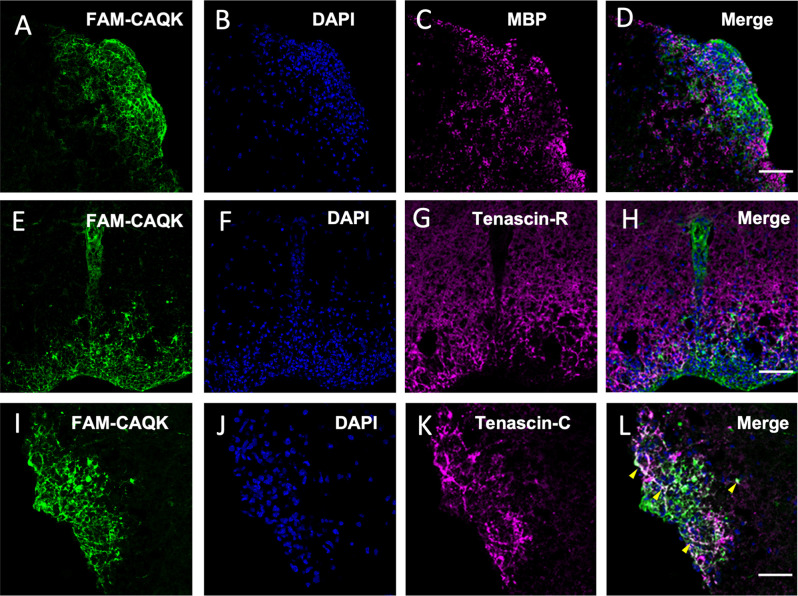
CAQK and tenascins in EAE lesions. **(A–D)** Confocal images identify FAM-CAQK peptide (**A,E,I**, green) at DAPI^+^ lesion clusters (**B,F,J**, blue) in the lumbar spinal cord of mice with EAE. The peptide does not associate with damaged myelin stained for MBP (**C**, magenta; merge in **D**) and largely complements staining for tenascin-R (**G**, magenta; merge in **H**). Some overlap is detected between FAM-CAQK and fibrous tenascin-C deposition (**K**, magenta) as indicated by the white signal in the merged image (**L**, arrowheads). Note that the majority of the lesion site covered by the peptide is negative for either tenascin **(H,L)**. Scale bars: 20 μm.

Reactive astrocytes contribute to changing the extracellular milieu at CNS injury sites (Roll and Faissner, 2019). The ECM molecule tenascin-C is absent in the healthy adult CNS but upregulated and deposited by reactive astrocytes at CNS injury sites (Roll and Faissner, 2019). Tenascin-R, expressed by astrocytes and oligodendrocytes in the adult CNS also undergoes extensive remodulation during CNS injury (Zhao et al., 2009). As CAQK was reported to bind components of an ECM complex, including tenascin-R, in the TBI model (Mann et al., 2016) and we consistently observed extracellular fibrous structures harboring the peptide, we next investigated FAM-CAQK association in combination with tenascins. First, staining for MBP showed in the EAE model that FAM-CAQK associates only marginally, if at all, with myelin membranes during demyelination (Figures 7A–D) similar to the findings in the cuprizone model (Figures 3D–F). Tenascin-R was detected in a uniform distribution over the uninjured spinal cord. In the illustrated example of demyelinating injury, identified by clusters of DAPI-stained mononucleated cells, tenascin-R deposits were mainly observed in healthy spinal cord tissue and appeared downregulated from the lesion area at the rim (Figures 7F,G). FAM-CAQK covered the cluster of DAPI^+^ cells at the lesion rim and accordingly showed no or only marginal overlap with tenascin-R (Figures 7E–H). Tenascin-C was selectively upregulated in interstitial spaces within DAPI^+^ EAE lesion sites and absent from surrounding healthy CNS tissue (Figures 7K,L). The FAM-CAQK signal overlapped in a fibrous pattern in regions of high tenascin-C accumulation and also covered a large region within the lesion that was devoid of tenascin depositions (Figures 7I–L). Thus, our data provide evidence for FAM-CAQK association with epitopes codistributing with tenascin-C, and simultaneously suggest additional yet unidentified peptide interactions with binding sites exposed on other cellular or extracellular molecules in demyelinating regions.

## Discussion

In this study, we report that the four amino acid peptide CAQK selectively targets demyelinating lesions. We show that upon systemic administration and 1 h in circulation, CAQK homes specifically to sites of CNS demyelinating injury and accumulates reliably and reproducibly at sites of myelin damage. We document these homing capacities in three different demyelination models, toxic, chemical, and inflammatory/autoimmune. Both cuprizone and lysolecithin-induced demyelination models are highly reproducible in the time course of demyelination with various degrees of BBB opening and neuroinflammation. CNS access of CAQK peptide through the compromised BBB was previously indicated from studies in the TBI model (Mann et al., 2016) and evident in the focal demyelination lysolecithin model. However, the suggestion that FAM-peptide can reach demyelinating lesions through the nearly closed endothelial barrier is novel. In the cuprizone model at the 6-week timepoint analyzed here, a tight BBB was documented in earlier studies (Bakker and Ludwin, 1987; Kondo et al., 1987; McMahon et al., 2002; Praet et al., 2014). Recent reports re-examining the BBB in the cuprizone model at early and late timepoints also confirmed almost intact barrier functions at the 6-week timepoint. These latter reports indicated compromised BBB permeability at the onset and during the first weeks of cuprizone feeding and progressive repair towards a nearly closed BBB at the 6-week timepoint (Berghoff et al., 2017; Shelestak et al., 2020). How CAQK enters the CNS and how it interacts with endothelial cells comprising the BBB requires detailed future studies that assess mechanisms in various models and coupled to different tags, therapeutic molecules, or targeting particles. The consistency of peptide targeting to lesion sites in three mouse models of demyelination suggests that CAQK is reliable as a targeting vector for demyelinating lesions in the CNS under diverse states of the BBB.

### CAQK targets components of the ECM

The extracellular matrix plays an important role during CNS development, homeostasis, and injury providing cues that regulate cell adhesion, signaling, and motility (Ruoslahti, 1996; Lau et al., 2013; Su et al., 2021). ECM components are secreted by resident cells and assemble into a scaffold in interstitial spaces to regulate a myriad of cellular responses. During white matter injury, the composition of the ECM dynamically changes as hyaluronan is proteolytically fragmented and CSPGs and tenascins are upregulated or remodeled (Lau et al., 2013; de Jong et al., 2020; Srivastava et al., 2020; Diao et al., 2021; Ghorbani and Yong, 2021). The altered ECM is inhibitory to OPC maturation, OPC recruitment to lesion sites, and remyelination (Franklin and Ffrench-Constant, 2008; Czopka et al., 2010).

In this light, it is significant that in the three demyelination models studied here, FAM-CAQK was targeted from the circulation to fibrous extracellular material at lesion sites. Original work from the Ruoslahti laboratory reported CAQK interactions with an extracellular matrix complex composed of versican, hyaluronan, proteoglycan link protein (Hapln), and tenascin-R by affinity purification of injured brain tissue and confirmed peptide colocalization by immunohistochemistry in the TBI model (Mann et al., 2016). Specific CAQK targeted epitopes within the lesioned ECM or its receptors, however, have remained elusive. In the current study, we chose to assess two ECMs, tenascin-R and tenascin-C that are dynamically regulated in white matter lesions and in TBI (George and Geller, 2018). In both the cuprizone and the EAE models at the time points examined, tenascin-R showed lower abundance at lesion sites than in healthy surrounding tissue, and accordingly, no mentionable peptide overlap with tenascin-R was observed. In contrast, tenascin-C deposits appeared to differing degrees within demyelinating lesions in the two models. In the EAE but not in the cuprizone model, CAQK localized to fibrous tenascin-C deposits suggesting that its target sites include epitopes in the local ECM. It is important to note, however, that most of the peptide targeted lesion area was devoid of tenascin-C expression. This leaves open the nature of the peptide interacting molecules at sites of demyelination and studies assessing peptide targeting during the dynamic progression of de-and remyelination will need to provide additional insights.

Our results are mixed regarding the question whether FAM-CAQK peptide homes to specific cellular targets. In none of the current models, the peptide associated with MBP+ demyelinating fibers at lesion sites, and contrary to findings in the TBI model (Mann et al., 2016) oligodendrocytes did not take up the peptide. At gross view, we find populations of reactive astrocytes associated the peptide in all three demyelination models. While our data cannot distinguish the cellular or pericellular distribution of the peptide with reactive astrocytes in the spinal cord models, FAM-CAQK seemed to reside in the cytoplasm of GFAP+ glia in the non-inflammatory cuprizone model (Figure 3). If confirmed, this could suggest autophagic activity during the 1 h in the circulation. In the inflammatory EAE model, CD3+ immune cells and Iba1+ microglia that enter the spinal cord parenchyma to initiate demyelinating lesions sequestered FAM-CAQK peptide from the circulation. Thus, the CAQK peptide associated with cell populations characterizing sites of injury supporting the conclusion that the peptide targets sites of demyelinating injury. Further work will be required to understand the nature of the peptide’s interactions with cells and whether phagocytosis of peptide-associated interstitial components by reactive astrocytic cells and microglia or other parameters are at play.

### Potential use of CAQK peptide to achieve remyelination after injury

Remyelination of demyelinated axons restores function and is important for preventing axonal and neuronal degeneration and thus functional and cognitive decline. The findings from the current study raise the possibility for translational purposes in diagnosis and therapeutic applications in aiding myelin repair. Because of its small size, CAQK is a candidate for biomedical imaging, for example for visualizing demyelinating lesions in MRI or PET scans, with CAQK coupled to ultra small super paramagnetic iron oxide nanoparticles (USPIO), or to radioactive tracers, respectively. These technologies are in development for the diagnosis of cancers (Wu et al., 2011; Novy et al., 2019; Rangger and Haubner, 2020), fibrosis (Belkahla et al., 2020), and detection of amyloid plaques in Alzheimer’s models (Wadghiri et al., 2013). Engineering CAQK peptides to adapt to medical imaging might aid earlier diagnosis of white matter lesions and demyelinating diseases.

A highly promising approach, already shown to work in animal models, is to couple the peptide to suitable transport carriers to target and deliver the therapeutic payload. In their original work, Ruoslahti and colleagues provided proof of this principle by targeting siRNA encapsulated in CAQK-coated porous nanoparticles to TBI lesions and locally suppressing gene expression for several days (Mann et al., 2016). Follow-up work in a spinal cord injury model engineered CAQK for exposure on mesenchymal stem cell-derived exosomes to deliver CRISPR/Cas9 plasmids suppressing the pro-inflammatory actions of tumor necrosis factor TNFα. CAQK-mediated targeting the injury site reduced inflammatory TNFα/NFκB signaling pathways, achieved tissue recovery, and restored motor function (Wang et al., 2022). Coupling of CAQK to nanoparticles could similarly enhance the targeting of therapeutic payloads to sites of demyelinating lesions thereby reducing toxicity and off-site effects (Liu et al., 2021). Neutralizing inhibitors of remyelination could enhance OPC recruitment and foster myelin repair, for example by blocking tenascins (Lau et al., 2013; Ghorbani and Yong, 2021; Su et al., 2021), ECM CSPGs (Lau et al., 2012; Keough et al., 2016), Hyaluronan (Back et al., 2005), Lingo-1 (Mi et al., 2008), or components of the Wnt and Notch pathways (John et al., 2002; Williams et al., 2007; Fancy et al., 2009; Syed et al., 2011). Conversely, several drugs including benztropine, clemastin, quetiapine, clobestasol, and minonazole show effects in improving OPC recruitment and remyelination (Deshmukh et al., 2013; Mei et al., 2014; Najm et al., 2015). Developing CAQK as a vector for targeting and concentrating such drugs at lesion sites may overcome the limits of systemic applications. Systemic drug administration often requires high concentrations and results in undesired side effects, thus packaging therapeutic drugs into peptide-coated natural or artificial particles could increase accumulation at lesion sites and improve myelin repair. Importantly, the CAQK binding epitope is preserved in human injured brain tissue (Mann et al., 2016) which provides an incentive to explore further applications for clinical utilization.

Demyelination is a hallmark of multiple sclerosis and loss of myelin leads to neurodegeneration. White matter abnormalities are evident in multiple neurodegenerative disorders, including Alzheimer’s and Parkinson’s disease and stroke (Chen et al., 2022 for review). With more refined detection methods, maintaining or restoring myelin functions at early stages of the disease may become a promising approach to halt neurodegenerative processes and physical and cognitive decline. By demonstrating CAQK targeting to myelin injuries and sites of progressive demyelination, the work reported here opens new possibilities for potential CAQK applications in early detection and diagnosis of MS and other neurodegenerative disorders and may ultimately prove useful to initiate myelin repair processes to limit neurodegeneration.

## Data Availability Statement

The original contributions presented in the study are included in the article/Supplementary material, further inquiries can be directed to the corresponding author.

## Ethics Statement

The animal study was reviewed and approved by Sanford Burnham Prebys Medical Discovery Institute Animal Care and Use Committee (La Jolla, CA). Written informed consent was obtained from the owners for the participation of their animals in this study.

## Author Contributions

Conceptualization, overall study design, organization of work and manuscript (BR); design and performance of experiments (CA-G: LPC and EAE models; DJ and YK: cuprizone model); manuscript writing or editing (all authors); manuscript revision (BR, CA-G, and DJ). All authors contributed to the article and approved the submitted version.

## Funding

This work was supported by a grant from the Office of Extramural Research, National Institute of Health R21 NS098400 to BR (provided salary support for CA-G, supplies and animals for LPC and EAE experiments and a portion of BR’s salary), R01NS103940 to YK (funded supplies and animals for cuprizone experiments), and TR01 AG71465 to JC (provided support for postdoc DJ).

## Footnotes

^1^ http://imagej.nih.gov/ij/index.html

## Supplementary Material

SUPPLEMENTARY FIGURE 1 FAM-CAQK peptide does not target normal myelin. In mice fed a normal diet. **(A,E)** FAM-CAQK peptide (green) does not associate with healthy myelin in corpus callosum (marked by dashed lines). Absence of Iba+ microglia (**B**, magenta) and the presence of myelin basic protein (**F**, MBP, magenta) indicate normal myelin. Merged images are shown in **(D,H)**, respectively. Panels **(C,G)** show DAPI-stained nuclei (blue). Circulating FAM-CAQK peptide reaches ependymal cells (Ep) lining the ventricle. Images were taken on a Zeiss Imager M2.

## Abbreviations

BBB: blood-brain barrier
BSA: bovine serum albumin
CAQK: cysteine-alanine-glutamine-lysine
CFA: complete Freund’s adjuvant
CNS: central nervous system
CSPG: chondroitin sulfate proteoglycan
DAPI: 4’,6-diamidino-2-phenylindole
EAE: experimental autoimmune encephalomyelitis
ECM: extracellular matrix
FAM: fluoresceinamine
FITC: fluorescein isothiocyanate
GFAP: glial fibrillary acidic protein
Hapln: proteoglycan link protein
Iba1: ionized calcium binding adaptor molecule 1
i.p.: intraperitoneally
LPC: lysophophatidylcholine (lysolecithin)
MBP: myelin basic protein
MRI: magnetic resonance imaging
MS: multiple sclerosis
NF-κB: nuclear factor kappa B
OCT: optimal cutting temperature compound
OPC: oligodendrocyte progenitor cell
PBS: phosphate-buffered saline
PET: positron emission tomography
PFA: paraformaldehyde
PID: post *induction* day
PTX: pertussis toxin
TBI: traumatic brain injury
TBST: Tris-buffered saline with 0.02% Triton X100
TNF: tumor necrosis factor
USPIO: ultrasmall super paramagnetic iron oxide nanoparticles.
