# Supplementary figures and images for: CAQK, a peptide associating with extracellular matrix components targets sites of demyelinating injuries
Source: Front Cell Neurosci. 2022 Aug 22;16:908401. doi: 10.3389/fncel.2022.908401 (PMC9441496; doi:10.3389/fncel.2022.908401)

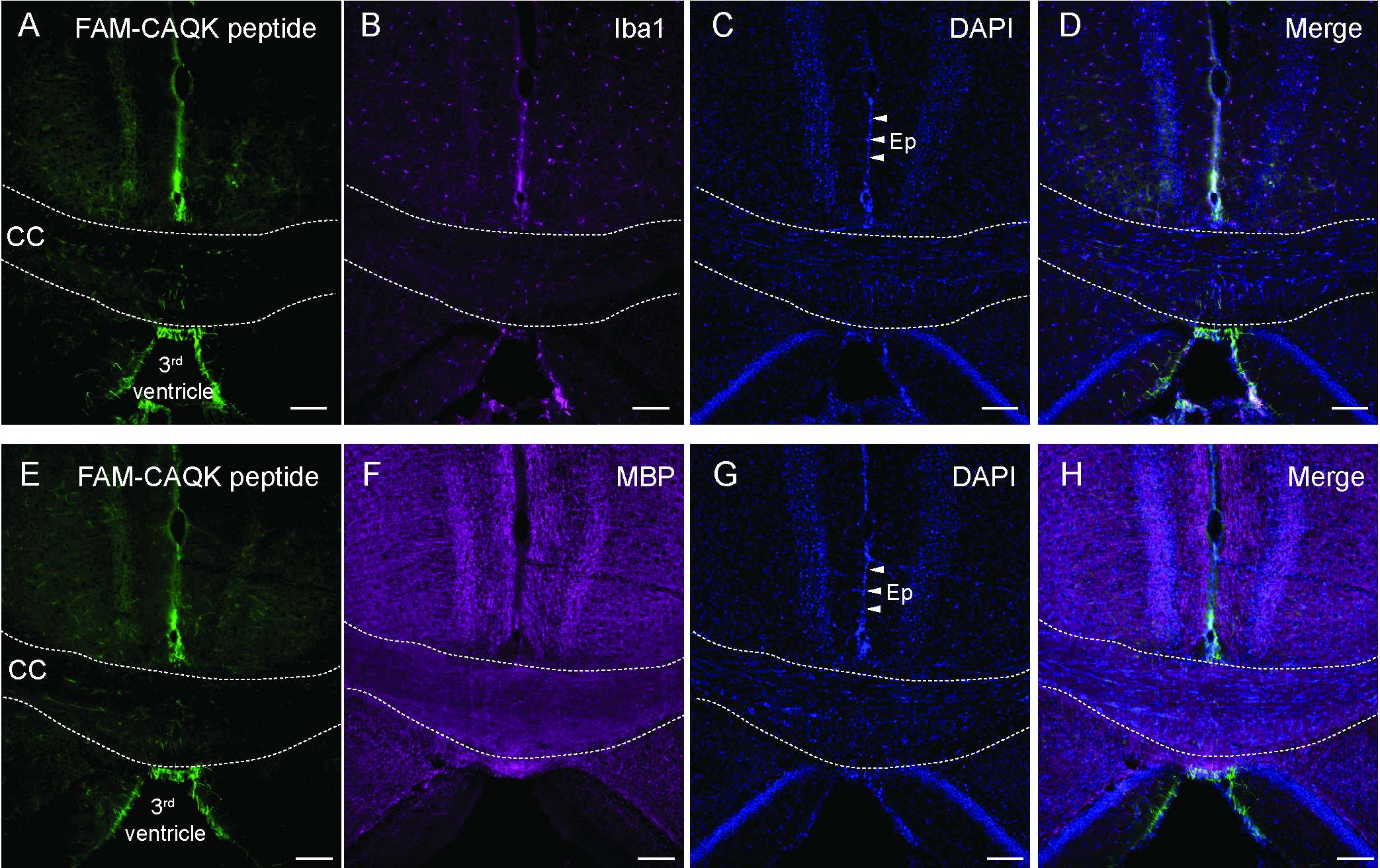

Supplement: SUPPLEMENTARY FIGURE 1 — FAM-CAQK peptide does not target normal myelin. In mice fed a normal diet. (A,E) FAM-CAQK peptide (green) does not associate with healthy myelin in corpus callosum (marked by dashed lines). Absence of Iba+ microglia (B, magenta) and the presence of myelin basic protein (F, MBP, magenta) indicate normal myelin. Merged images are shown in (D,H), respectively. Panels (C,G) show DAPI-stained nuclei (blue). Circulating FAM-CAQK peptide reaches ependymal cells (Ep) lining the ventricle. Images were taken on a Zeiss Imager M2. [file Image_1.tif]
